# Clinical outcomes of reverse shoulder arthroplasty in obstetric brachial plexus palsy with suprascapular nerve injury and glenohumeral osteoarthritis

**DOI:** 10.1016/j.jcot.2025.103096

**Published:** 2025-06-09

**Authors:** F. Granata, S. D'Amato, S. Cassaro, C. Burgio, F. Bosco

**Affiliations:** a Department of Orthopaedics and Traumatology, G.F. Ingrassia Hospital Unit, ASP 6, Palermo, Italy; b Adult Reconstruction and Joint Replacement Service, Hospital for Special Surgery, New York, NY, USA; c Department of Precision Medicine in Medical, Surgical and Critical Care (Me.Pre.C.C.), University of Palermo, Palermo, Italy

**Keywords:** Brachial plexus neuropathies, Shoulder prosthesis, Arthroplasty, Replacement, Shoulder joint, Treatment outcome

## Abstract

**Background:**

Untreated obstetric brachial plexus palsies (OBPP) present with various upper limb impairments, including muscle weakness, soft tissue contractures, and osteoarticular deformities. Reverse shoulder arthroplasty (RSA) medializes the center of rotation, optimizing deltoid function to compensate for deficits caused by OBPP. This study evaluates the effectiveness of RSA in patients with untreated OBPP involving the suprascapular nerve and end-stage glenohumeral osteoarthritis (OA), focusing on functional and quality-of-life improvements.

**Methods:**

A retrospective analysis was performed on patients with OBPP involving the suprascapular nerve and end-stage glenohumeral OA who underwent RSA. Included patients had significant deficits in the supraspinatus and infraspinatus muscles but preserved deltoid function. Pre- and postoperative assessments included Range of Motion (ROM), Visual Analog Scale (VAS), and Patient-Reported Outcome Measures (PROMs). Data on complications and revision surgeries were also collected.

**Results:**

Seven patients (2 males, 5 females; mean age 38.9 ± 14.9 years) with a median follow-up of 3.1 ± 2.2 years were included. Postoperatively, significant improvements were observed in limb function and quality of life, as reflected in the Constant Shoulder Score (CSS), Oxford Shoulder Score (OSS), and Quick Disabilities of the Arm, Shoulder, and Hand (QDASH). Pain levels decreased markedly, and ROM showed a moderate increase. No complications or revision surgeries were reported.

**Conclusions:**

RSA is an effective and safe surgical option for patients with untreated OBPP involving the suprascapular nerve and end-stage glenohumeral OA, yielding significant functional and quality-of-life improvements at short- and mid-term follow-up. The absence of complications and revision surgeries further supports its reliability in this challenging patient population.

**Level of evidence:**

IV.

## Graphical abstract

**Figure:**
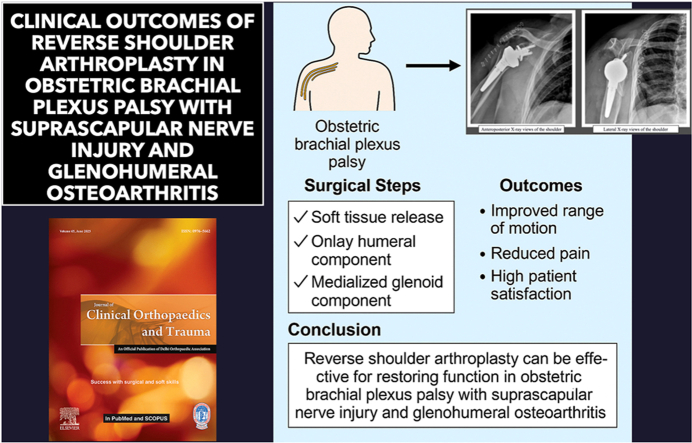

## 1 Introduction

Obstetric brachial plexus injuries (OBPI), commonly known as obstetric brachial plexus palsy (OBPP), represent a spectrum of clinical conditions characterized by paralysis or weakness of the upper limb.^1^, ^2^, ^3^ These injuries typically result from excessive traction on the affected limb during childbirth, whether in standard or complicated deliveries, causing damage to the brachial plexus.^1^^,^^2^^,^^4^, ^5^, ^6^, ^7^, ^8^ The most common presentation involves injury to the C5 and C6 nerve roots, called Erb-Duchenne palsy. At the same time, a less frequent variant affects the lower trunk of the brachial plexus (C7-C8-T1), termed Dejerine-Klumpke palsy.^4^, ^5^, ^6^^,^^9^, ^10^, ^11^

The consequences of untreated OBPP during neonatal and early childhood stages are often severe and irreversible. Patients may develop significant functional impairments, including severe restrictions in shoulder range of motion (ROM), progressive glenohumeral joint deformities, muscle atrophy, and soft tissue contractures.^12^, ^13^, ^14^, ^15^, ^16^ These complications compromise the structural integrity of the shoulder joint but also the functional capacity of the upper limb, adversely affecting the individual's ability to perform daily activities and substantially reducing their quality of life.^2^^,^^3^^,^^7^^,^^10^^,^^17^, ^18^, ^19^, ^20^

Untreated OBPP remains a particularly challenging condition to manage due to the complexity of its long-term outcomes, necessitating the exploration of therapeutic strategies to address its debilitating effects.^10^, ^11^, ^12^, ^13^^,^^20^, ^21^, ^22^, ^23^, ^24^ Among available surgical interventions, reverse shoulder arthroplasty (RSA) implantation has emerged as a promising solution to address functional deficits associated with OBPP. This approach is particularly relevant for glenohumeral osteoarthritis (OA) cases and significant muscle deficits.^3^^,^^11^^,^^12^^,^^22^ RSA leverages the deltoid muscle to compensate for impaired rotator cuff function, offering potential improvements in limb functionality and quality of life. Nevertheless, RSA's clinical and functional outcomes in this population remain underexplored, with limited evidence supporting its long-term effectiveness.^3^^,^^11^^,^^12^^,^^22^

The aim of this study is to assess the clinical and functional outcomes of RSA implantation in patients with untreated OBPP involving the suprascapular nerve and end-stage glenohumeral OA. Specifically, the study evaluates pain levels, ROM, and patient-reported quality of life improvements. The hypothesis is that RSA implantation may provide significant enhancements in functional outcomes and quality of life for these patients by restoring a functional center of rotation in the shoulder joint and reducing pain.

## 2 Material and methods

### 2.1 Study design

This retrospective study analyzed a consecutive series of seven patients diagnosed with obstetric brachial plexus palsy (OBPP) presenting as Erb-Duchenne palsy involving the suprascapular nerve and end-stage glenohumeral OA. The condition was classified according to the Walch classification system.^14^ All patients underwent primary RSA (SMR® RSA, LimaCorporate, Udine, Italy) at a single orthopedic referral center, between 2017 and October 2023.

### 2.2 Inclusion and exclusion criteria

Inclusion criteria included a diagnosis of untreated OBPP with end-stage glenohumeral osteoarthritis, significant supraspinatus and infraspinatus deficits, and preserved electromyographic activity of the deltoid muscle. Additional inclusion criteria were age ≥18 years and a minimum follow-up of one year.

The exclusion criteria excluded patients with compromised deltoid muscle function, previous complex trauma-related surgical procedures, or failure to adhere to postoperative rehabilitation protocols.

### 2.3 Surgical technique

Preoperative radiographic planning was performed using standard anteroposterior and lateral X-ray views of the shoulder. An experienced shoulder surgeon (F.G.) carried out the surgical procedure. The patient was positioned in the beach chair position, and a deltopectoral approach was utilized. Following careful dissection of the deltopectoral fascia, the underlying structures were exposed, including the cephalic vein and the long head of the biceps tendon (LHBT), which were identified and isolated. The LHBT was secured with No. 2 Vycril® (Ethicon, Somerville, New Jersey, USA) and severed from its proximal insertion, leaving the suture needle in place for potential reinsertion.

The humeral head was dislocated, and the glenoid cavity was prepared to accommodate the prosthesis's glenoid component. A metal-backed baseplate with a polyethylene glenosphere insert was implanted into the prepared glenoid surface and stabilized with a central screw. The humeral head was then excised, and the humeral canal was prepared using reamers to ensure an appropriate fit for the prosthesis. A cementless stem was inserted into the humeral canal, followed by the placement of a dedicated humeral body and a metal reverse liner.

**Fig. 1:**
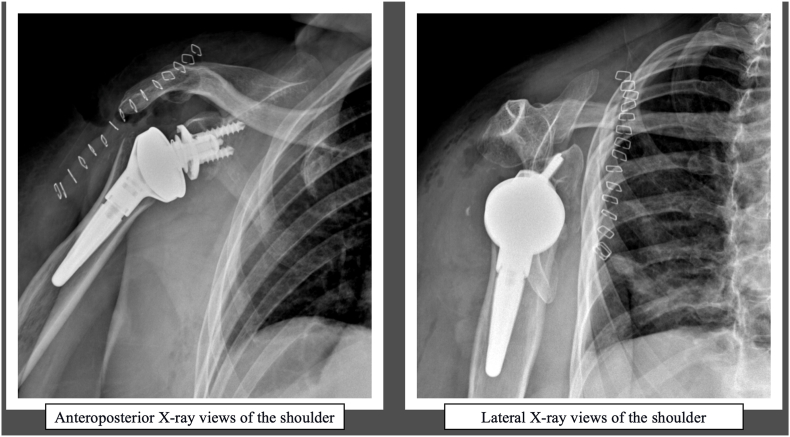
Anteroposterior (left) and lateral (right) X-ray views of the shoulder following reverse shoulder arthroplasty (RSA) performed as a treatment for obstetric brachial plexus palsy (OBPP). The images illustrate the positioning of the prosthesis and the reconstructed shoulder anatomy post-surgery.

The humeral component was reduced into the glenoid socket, effectively reversing the normal anatomy of the shoulder joint. After confirming the implant's stability and ROM, the incision was closed in layers. The deltoid and pectoralis major muscles were reapproximated, and the skin was closed using sutures or staples. Postoperative X-rays in anteroposterior and lateral views were taken to validate the adequacy of the reduction and assess the implant's stability (Fig. 1). All patients received a medialized glenoid and lateralized humeral configuration with a 145° onlay humeral stem (SMR® RSA, LimaCorporate, Italy), designed to optimize deltoid leverage in absence of cuff function. Preoperative CT scans were performed in 5 of 7 patients to assess glenoid version and bone stock. No augmented glenoid components were necessary.

### 2.4 Postoperative protocol

Following surgery, all patients received postoperative care, which included the use of a sling for up to four weeks, along with ice and compression therapy to minimize swelling.^4^^,^^15^ A standardized rehabilitation protocol was implemented, beginning with early mobilization through passive ROM and pendulum exercises. These exercises gradually progressed to improve shoulder mobility.

As tolerated, patients advanced to active-assisted ROM exercises, followed by active ROM exercises. Six to seven weeks postoperatively, resistance training with light weights or resistance bands was introduced, along with functional activities to enhance coordination and proprioception. Regular follow-up appointments were scheduled to monitor recovery progress and address any postoperative complications. The postoperative rehabilitation protocol did not differ from the standard RSA protocol and was applied uniformly.

### 2.5 Data extraction

Demographic and clinical information was systematically recorded using a standardized form. Collected data included sex, age at surgery, hand dominance, affected shoulder side, and length of follow-up. During the follow-up period, two authors (S.D. and S.C.) conducted data collection through direct patient examinations, patient-reported outcome measures (PROMs),^16^, ^17^, ^18^, ^19^ and clinical evaluation forms. Any postoperative complications were recorded and documented.

One author (C.B.) managed data analysis independently and was supervised by an experienced shoulder surgeon (F.G.) to resolve any uncertainties. A third author (F.B.) provided a resolution to disagreements.

### 2.6 Outcome evaluation

Clinical outcomes were assessed using the Visual Analog Scale (VAS),^19^ ROM measurements, the Quick Disabilities of the Arm, Shoulder, and Hand (QDASH) questionnaire,^18^ the Constant Shoulder Score (CSS),^17^ and the Oxford Shoulder Score (OSS).^16^

The VAS, a subjective measure of pain intensity, uses a 10-point scale ranging from “no pain” to “worst pain”.^19^ ROM assessments evaluated shoulder joint mobility, focusing on forward flexion, abduction, external rotation, and internal rotation. The CSS is a comprehensive tool that measures shoulder function, considering pain, activity level, range of motion, and strength, with a maximum score of 100 indicating optimal function.^17^

The QDASH evaluates the impact of upper limb musculoskeletal conditions on daily life, where higher scores represent greater disability.^18^ The OSS is a self-reported questionnaire designed to assess shoulder pain and function, with scores ranging from 12 (excellent function) to 60 (poor function).^16^ These measures comprehensively evaluated patient outcomes and quality of life post-surgery.

### 2.7 Ethical approval

This study was classified as exempt from Institutional Review Board (IRB) approval, as it involved a retrospective analysis of a well-established surgical procedure. The research was conducted strictly with the ethical principles outlined in the 1964 Declaration of Helsinki and its subsequent amendments.

### 2.8 Statistical analysis

The statistical evaluation was conducted with MedCalc (MedCalc Software, Ostend, Belgium). Continuous variables were analyzed through measures of central tendency and dispersion, while categorical variables were reported using absolute and relative frequencies. For normally distributed variables, differences between means were assessed using the Student's t-test; the Mann-Whitney *U* test was used for non-parametric data. Statistical significance was set at p < 0.05. Microsoft Excel facilitated Data collection and management (Version 2019, Microsoft, Redmond, WA).

## 3 Results

### 3.1 Demographics

Seven eligible patients (2 males, 5 females) were recruited and analyzed, each with a minimum follow-up duration of one year. The median follow-up duration was 3.1 ± 2.2 years. The mean age at the time of surgery was 38.9 ± 14.9 years. All prostheses were implanted on the right side, which represented the dominant shoulder in all but two patients. Comprehensive demographic and clinical data for all patients are summarized in Table 1.

**Table 1:** Demographic and clinical data of patients enrolled in the study cohort.

Patients	Age at the time of surgery (years)	Sex (M/F)	Dominant arm (R/L)	Side of surgery (R/L)	Follow-up (years)
N°	Mean ± SD (Range Min-Max)				Mean ± SD
Patient 1	18	M	R	R	7.1
Patient 2	38	F	R	R	1.7
Patient 3	53	F	L	R	2.8
Patient 4	23	M	R	R	5.3
Patient 5	41	F	L	R	1
Patient 6	39	F	R	R	1.5
Patient 7	60	F	R	R	2.6
Total	38.9 ± 14.9 (18–45)	2/5	5/2	7/0	3.1 ± 2.2

N°—number of evaluation cases; SD−standard deviation; %—percentage; R—right; L—Left; Min—minimum; Max—maximum.

### 3.2 Clinical evaluation

Preoperative assessments revealed typical OBPP features involving the suprascapular nerve and end-stage glenohumeral OA, with the shoulder in adduction and internal rotation. Electromyography showed good deltoid activity but severe weakness in the subscapularis and supraspinatus and marked infraspinatus deficits.

Postoperative evaluations demonstrated significant improvement in all ROM parameters. Active flexion increased from 85.6° ± 9.9°–116.6° ± 8.8° (p < 0.05). While external rotation, abduction, and internal rotation improved, changes were not statistically significant (Table 2).

**Table 2:** Preoperative and postoperative range of motion of patients enrolled.

Shoulder ROM	Mean	SD	*p* value
FL pre-operative	85.6	9.9	
FL post-operative	116.6	8.8	
Δ	31.0	2.7	*p* < 0.001
AB pre-operative	73.7	12.3	
AB post-operative	93.3	11.0	
Δ	19.6	6.0	*p* = 0.085
IR pre-operative	69.3	4.6	
IR post-operative	74.6	6.2	
Δ	5.3	2.6	*p* = 0.094
ER pre-operative	10.9	4.0	
ER post-operative	15.0	3.6	
Δ	4.1	2.0	*p* = 0.067

ROM—Range of motion; FL—Forward Flexion; AB—Abduction; IR—Internal Rotation; ER—External Rotation; PRE—Pre-operative; POST: Post-operative; Δ: Difference Post-operative ROM - Pre-operative ROM; SD = standard deviation; p: p-value.

At final follow-up, all clinical outcome scores improved significantly (p < 0.05). VAS pain scores dropped from 8.4 ± 0.5 to 3.3 ± 1.7, consistent with previous reports.^3^^,^^12^^,^^13^ OSS,^16^ CSS,^17^ and QDASH^18^ also showed marked improvement (Table 3).

**Table 3:** Preoperative and postoperative clinical outcomes of patients enrolled.

Clinical outcomes	Mean	SD	*t*	*p*
CSS pre-operative	22.4	2.4	11.091	<0.0001
CSS post-operative	53.4	6.5		
QDASH- pre-operative	35.9	3.4	48396	0.0004
QDASH- post-operative	22.3	6.6		
OSS- pre-operative	39.3	1.4	7.9375	<0.0001
OSS- post-operative	20.1	6.2		
VAS- pre-operative	8.4	0.5	7.6177	<0.0001
VAS- post-operative	3.3	1.7		

SD = standard deviation; CSS = Constant Shoulder Score; QDASH = Quick Disability of the Arm, Shoulder and Hand; OSS = Oxford Shoulder Score; VAS = Visual Analog Scale; *t* = Student's t-test; *p*: p-value.

### 3.3 Patient satisfaction

Of the seven patients, six reported being satisfied with the surgical procedure and stated they would undergo the surgery again if necessary. However, one patient was not satisfied, reporting persistent pain comparable to preoperative levels and an unsatisfactory recovery in mobility, falling short of expectations.

### 3.4 Complications

No short- or medium-term complications were observed during the outpatient follow-up period, reinforcing the procedure's safety profile in this cohort.

## 4 Discussion

The main finding of this study is that RSA is an effective surgical intervention for patients with untreated OBPP involving the suprascapular nerve and end-stage glenohumeral OA during neonatal and early childhood stages. RSA demonstrated promising clinical outcomes with significant improvements in pain reduction and overall patient satisfaction at short- and medium-term follow-up. Importantly, no complications or revision surgeries were recorded in the studied cohort, highlighting the safety of this approach. The improvement in PROMs, including the VAS, OSS, CSS, and QDASH, aligns closely with findings from existing literature.^3^^,^^11^^,^^12^ Furthermore, patients reported satisfaction with their outcomes, particularly in terms of pain reduction and modest gains in shoulder ROM.

OBPP typically results from traction injury during delivery, with most cases resolving spontaneously.^4^^,^^5^^,^^20^ However, incomplete recovery can lead to persistent deficits, soft tissue contractures, and joint deformities,^10^^,^^20^ requiring surgical management in severe cases of glenohumeral dysplasia and OA.^10^^,^^23^

While hemiarthroplasty (HA) and total shoulder arthroplasty (TSA) are established options—HA preserving the glenoid, TSA replacing both components—TSA generally offers better pain relief and lower revision rates, though without superior ROM gains.^23^ In a case report by Gosens et al.,^24^ HA in Erb's palsy initially improved function but eventually required revision, with suboptimal results.

Linked-constrained TSA provides mechanical stability through a fixed connection between components but increases stress at the bone-implant interface, with a higher risk of complications. Rudge et al.^13^ reported satisfactory pain relief but minimal ROM gains and a high complication rate (fracture, three revisions) in nine Erb's palsy patients treated with constrained TSA.

In contrast, RSA offers a biomechanical advantage in OBPP-related muscle deficits by medializing the center of rotation, enabling the deltoid to compensate for rotator cuff insufficiency. This makes RSA particularly suited for OBPP patients with supraspinatus and infraspinatus dysfunction.

Existing literature supports RSA's effectiveness in improving function in patients with OBPP and glenohumeral OA. Porcellini et al.^3^ reported significant gains in flexion (91.25°–122.55°) and external rotation (11.25°–17.5°) in four patients. Werthel et al.^12^ compared outcomes of HA, TSA, and RSA in six patients, finding that only RSA yielded forward flexion above 90°, with one case reaching 140°, highlighting its superior potential for restoring ROM.

However, outcomes depend on patient selection and preoperative conditions. In our cohort, despite selecting patients without deltoid impairment, ROM improvements remained modest. Persistent limitations in elevation, abduction, and rotation likely stem from chronic muscle hypotrophy and contractures, as confirmed by EMG findings of severe deficits.

Pain reduction and functional improvement are well supported. Porcellini et al.^3^ showed a drop in OSS from 48.8 to 18.3. Kurowicki et al.^11^ reported total pain relief (VAS = 0) after RSA in a case of brachial plexopathy. Werthel et al.^12^ observed VAS scores improving from 9 to 4 (HA/TSA) and 10 to 4 (RSA), favoring RSA slightly in pain control.

Regarding our cohort of patients, one of the seven patients treated with RSA implantation expressed dissatisfaction due to persistent pain and limited improvement in ROM, which may be attributed to insufficient release of musculo-tendinous structures during surgery. Specifically, inadequate management of retracted tissues, such as the conjoint tendon, could have contributed to these outcomes.^25^ This highlights the critical importance of thorough preoperative evaluation and surgical planning to address soft tissue retractions and imbalances associated with OBPP sequelae.^25^^,^^26^ Specifically, appropriate release of contracted structures such as the conjoint tendon is crucial to maximize mobility and avoid functional limitations. Future surgical protocols should emphasize the careful release of musculo-tendinous structures to optimize outcomes and prevent similar cases of dissatisfaction.

Despite these challenges, no complications or revision surgeries were observed during follow-up, aligning with previous reports.^3^^,^^11^^,^^12^ This supports the safety and feasibility of RSA as a treatment option for OBPP-related glenohumeral osteoarthritis, even in the presence of complex anatomical alterations.

Therefore, RSA represents a promising surgical option for managing functional deficits and pain in patients with OBPP and glenohumeral OA. While RSA demonstrates clear advantages over HA and TSA in certain cases, achieving optimal outcomes requires careful preoperative planning and patient selection. Future studies should focus on strategies to address the limitations posed by long-standing muscle hypotrophy and soft tissue contractures, which remain significant barriers to achieving full functional recovery in this patient population.

The main strength of this study is its demonstration of the efficacy of reverse RSA in patients with untreated OBPP involving the suprascapular nerve and end-stage glenohumeral OA. At short- and mid-term follow-up, RSA significantly reduced pain and moderately improved function, with no revisions required. These findings support RSA as a safe and reliable option for managing complex OBPP-related sequelae, even in anatomically challenging cases. The absence of complications further underscores the procedure's safety profile.

This study has several limitations. First, its retrospective nature may introduce selection bias and variability in data quality, limiting control over confounding factors and potentially impacting reliability. Second, the small sample size (n = 7) reduces statistical power and limits generalizability, particularly regarding rare complications. Further research involving larger cohorts is necessary to validate these results and strengthen their external applicability. Third, being a single-center study, findings may not be applicable to other settings due to variations in surgical technique, rehabilitation, and patient demographics. Future multicenter, prospective studies with standardized protocols and extended follow-up are necessary to validate these results and better assess the long-term effectiveness of RSA in managing OBPP-related complications, including sustained pain relief, functional gains, and implant survival.

## 5 Conclusion

This study demonstrates that adopting RSA in patients with untreated OBPP involving the suprascapular nerve and end-stage glenohumeral OA is a reliable treatment, achieving significant improvement in pain symptoms and moderate improvement in daily activities. Moreover, no complications or revision procedures were recorded in the short-to mid-term follow-up. Nonetheless, despite high patient-reported satisfaction, functional improvement of the limb remained limited, likely due to persistent rotator cuff muscle hypotrophy. Further studies are needed to validate these results and to assess the long-term effectiveness and durability of RSA in patients with untreated OBPP.

## CRediT authorship contribution statement

**F. Granata:** contributed to the study conception and design. Material preparation and data collection were performed by, assessed the scientific contents and the writing. **S. D'Amato:** Data analyses were performed by, The first draft of the manuscript was written by. **S. Cassaro:** Data analyses were performed by, and all authors commented on previous versions of the manuscript. **C. Burgio:** and all authors commented on previous versions of the manuscript. **F. Bosco:** contributed to the study conception and design. Material preparation and data collection were performed by, The first draft of the manuscript was written by, and all authors commented on previous versions of the manuscript, assessed the scientific contents and the writing. All authors read and approved the final manuscript.

## Guardian/patient's consent

Not Applicable.

## Ethics approval

Compliant with Helsinki, retrospective, exempt from IRB.

## Consent

Written informed consent obtained.

## Data availability

Available on request.

## Code availability

Not applicable.

## Ethical statement

This study was conducted in accordance with the ethical standards of the institutional and/or national research committee and with the 1964 Helsinki Declaration and its later amendments or comparable ethical standards.

## Funding STATEMENT

No funding has been received for this study.

## Conflict of interest statement

On behalf of all authors, the corresponding author states that there is no conflict of interest.
